# Aripiprazole as second-line pharmacotherapy for a heterogeneous cohort of refractory functional dizziness disorders: a pilot preliminary retrospective study

**DOI:** 10.3389/fneur.2026.1858965

**Published:** 2026-06-03

**Authors:** Fumiyuki Goto, Koichiro Wasano

**Affiliations:** 1 Department of Otolaryngology-Head and Neck Surgery, Tokai University School of Medicine, Isehara, Kanagawa, Japan; 2 Goto ENT & Dizziness Clinic, Machida, Tokyo, Japan

**Keywords:** aripiprazole, functional dizziness, persistent postural-perceptual dizziness, refractory dizziness, second-line pharmacotherapy, serotonin-dopamine modulator

## Abstract

**Background and objective:**

First-line serotonergic antidepressants achieve clinical response in approximately 60–65% of patients with persistent postural-perceptual dizziness (PPPD), leaving a substantial refractory population. Aripiprazole, a serotonin-dopamine modulator with partial D2 and 5-HT1A agonism and 5-HT2A antagonism, may address dopaminergic and emotional dysregulation insufficiently controlled by serotonergic agents alone. This study evaluated the preliminary efficacy and tolerability of aripiprazole as second-line pharmacotherapy in patients with refractory functional dizziness disorders, including PPPD.

**Methods:**

We retrospectively reviewed 16 patients (6 men, 10 women; mean age 50 ± 16.1 years) with refractory dizziness disorders, including PPPD (*n* = 9), psychogenic dizziness (*n* = 4), vestibular migraine (*n* = 2), and Ménière's disease (*n* = 1), who received aripiprazole (1.5–3.0 mg/day) after insufficient response to first-line antidepressants. The primary outcome was treatment response based on the Clinical Global Impression-Improvement scale (CGI-I; score 1–3 defined as improvement). Secondary outcomes included changes in Dizziness Handicap Inventory (DHI), Niigata PPPD Questionnaire (NPQ), and foam posturography in evaluable patients.

**Results:**

CGI-I response was observed in 12 of 16 patients (75.0%). Four patients were non-responders, and one discontinued treatment because of extrapyramidal adverse effects. In patients with available questionnaire data (*n*=4–5), DHI and NPQ scores improved after treatment. Foam posturography showed no significant changes.

**Conclusion:**

In this pilot retrospective series, aripiprazole demonstrated promising preliminary efficacy as a second-line treatment for refractory functional dizziness disorders. Its multimodal serotonin-dopamine mechanism may target symptoms insufficiently controlled by serotonergic agents alone. Given the small sample size and uncontrolled design, these findings should be considered hypothesis-generating. Prospective controlled studies are warranted.

## Introduction

Persistent postural-perceptual dizziness (PPPD) is the most prevalent chronic vestibular disorder in specialized dizziness clinics, affecting an estimated 20% of patients presenting with chronic dizziness (1). Defined by the Barany Society in 2017, PPPD is characterized by non-spinning dizziness, unsteadiness, or subjective spatial disorientation persisting for >= 3 months, exacerbated by upright posture, active or passive motion, and complex visual stimuli (1). Its pathophysiology involves dysregulated sensory weighting and cortical threat-appraisal circuits, with frequent comorbidity of anxiety and depression (2).

Serotonergic antidepressants—primarily selective serotonin reuptake inhibitors (SSRIs) and serotonin-norepinephrine reuptake inhibitors (SNRIs)—are the established first-line pharmacotherapy for PPPD (3). However, their overall response rate is approximately 60–65% (3, 4), leaving a substantial proportion of patients with refractory symptoms despite adequate treatment. A landmark longitudinal study published in Frontiers in Neurology demonstrated that serotonergic antidepressants produce sustained improvements in Dizziness Handicap Inventory (DHI), Hospital Anxiety and Depression Scale (HADS), and Niigata PPPD Questionnaire (NPQ) scores over 3 years in PPPD patients, yet somatosensory hypersensitivity—a key pathophysiological feature in a subset of patients—did not normalize with pharmacotherapy alone (4). These findings highlight both the efficacy and the ceiling effect of serotonergic agents, underscoring the unmet need for additional pharmacological strategies.

Aripiprazole is an atypical antipsychotic classified as a serotonin-dopamine modulator (SDM). Its pharmacological profile includes partial agonism at dopamine D2 and D3 receptors, partial agonism at serotonin 5-HT1A receptors, and antagonism at 5-HT2A receptors (5). This multimodal mechanism normalizes dopaminergic tone bidirectionally—increasing activity in hypodopaminergic states and reducing it in hyperdopaminergic states—while simultaneously modulating serotonergic circuits. Given that dopamine plays a central role in emotional regulation, reward processing, and motivational salience, aripiprazole may specifically target the affective dysregulation that underlies treatment resistance in PPPD patients who have failed serotonergic monotherapy.

We have previously published case reports documenting the efficacy of aripiprazole in individual patients with refractory PPPD and related functional dizziness disorders (6, 7). Building on these observations, the present study aimed to provide preliminary retrospective evidence on the efficacy and tolerability of aripiprazole as second-line pharmacotherapy across a larger cohort of patients with refractory dizziness including PPPD.

## Method

This was a single-center retrospective observational study conducted at Goto ENT & Dizziness Clinic (Machida, Tokyo, Japan). Medical records were reviewed for patients who met all of the following criteria: (1) a clinical diagnosis of PPPD according to Barany Society 2017 criteria (1) or other functional/refractory dizziness disorder (psychogenic dizziness, vestibular migraine, or Meniere's disease); (2) demonstrated insufficient clinical response to at least one first-line antidepressant (SSRI, SNRI, noradrenergic and specific serotonergic antidepressant [NaSSA], tricyclic antidepressant [TCA], or multimodal antidepressant) administered for a minimum of 4 weeks at a clinically adequate dose. For the purpose of this study, “refractory dizziness” was operationally defined as the persistence of clinically significant dizziness symptoms causing continued functional impairment in daily activities (Dizziness Handicap Inventory total score >=30 or equivalent clinician judgment of moderate-to-severe handicap) together with a treating-clinician assessment of no clinically meaningful improvement (CGI-Improvement score >=4) after the first-line antidepressant trial described above. Exclusion criteria were: previous intolerance or known contraindication to aripiprazole, pregnancy or lactation, and presence of a primary psychiatric disorder under active specialist psychiatric care; and (3) subsequently treated with aripiprazole (1.5–3.0 mg/day) either as augmentation to ongoing antidepressant therapy or as replacement monotherapy. Treatment Protocol. Aripiprazole was initiated at 1.5 mg once daily, administered orally in the evening to minimize daytime sedation. After 2–4 weeks at the starting dose, the dose was up-titrated to 3.0 mg/day in patients who showed partial response with acceptable tolerability; patients who achieved satisfactory response at 1.5 mg/day were maintained at that dose. The maximum dose in this series was 3.0 mg/day. The choice between augmentation (continuation of the prior antidepressant at its existing dose) and replacement monotherapy (tapering of the prior antidepressant over 2–4 weeks prior to or concurrent with aripiprazole initiation) was made at the treating clinician's discretion based on prior tolerability, residual symptom profile, and patient preference. Treatment response was assessed at the routine follow-up visit occurring at a minimum of 8 weeks after aripiprazole initiation; mean treatment duration at the time of CGI-I assessment was 16.4 weeks (range 8–32 weeks). Patients who showed no response or intolerance during this period were classified as treatment failures and aripiprazole was discontinued. The study was approved by the institutional ethics committee (Approval No. E2026-02-001, February 2, 2026). Informed consent was waived given the retrospective design.

Diagnostic criteria for non-PPPD disorders and concomitant disease-specific therapy. Diagnoses of non-PPPD disorders were assigned by the treating otologist using established criteria, as follows. Psychogenic dizziness was diagnosed when the patient presented with persistent dizziness in the absence of an identifiable peripheral or central vestibular lesion on standardized otoneurological evaluation (caloric testing or videonystagmography, video head-impulse test, pure-tone audiometry, and brain MRI when clinically indicated), and when symptoms were judged by the treating clinician to be clearly precipitated, maintained, or exacerbated by an identifiable psychological stressor or comorbid psychiatric condition (such as a depressive or anxiety disorder), while not fully meeting the Barany Society criteria for PPPD. Vestibular migraine was diagnosed according to the Barany Society and International Headache Society consensus criteria (2012); all patients with vestibular migraine had been receiving, and continued to receive throughout aripiprazole treatment, standard prophylactic antimigraine pharmacotherapy (lomerizine, propranolol, or topiramate, individualized at the discretion of the treating clinician) for a minimum of 3 months prior to aripiprazole initiation. Meniere's disease was diagnosed according to the Barany Society 2015 criteria; the included Meniere's patient had received and continued betahistine together with a diuretic (isosorbide) and dietary salt restriction for more than 6 months prior to aripiprazole initiation. For vestibular migraine and Meniere's disease patients, “refractory” was specifically defined as persistence of clinically significant non-vertiginous interictal dizziness or imbalance with continued functional impairment, despite both optimal disease-specific therapy as outlined above and the first-line antidepressant trial described in inclusion criterion (2). With respect to non-pharmacological interventions for PPPD, vestibular rehabilitation exercises were offered to all PPPD patients as part of routine outpatient care; however, adherence and frequency varied between patients and were not systematically recorded. Formal cognitive-behavioral therapy was not available within our clinic setting and was not undertaken by any patient prior to aripiprazole initiation. The implications of this constraint are addressed in the Limitations.

The primary outcome was clinical treatment response, defined as a CGI-I (Clinical Global Impression-Improvement) score of 1–3 (very much improved, much improved, or minimally improved) at the clinician's final assessment. The CGI-I is a validated 7-point clinician-rated scale widely used in pharmacological trials of functional neurological and psychiatric disorders. Secondary outcomes included, in a subset of patients for whom data were available: pre- and post-treatment scores on the DHI, the NPQ (8), and the Hospital Anxiety and Depression Scale (HADS); and foam posturography (closed-eye sway area, cm2) as an objective measure of postural stability. Given the small and incomplete nature of the secondary dataset, formal statistical analysis was not performed for secondary outcomes.

## Results

Sixteen patients were included. Patient characteristics and CGI-I response rates by diagnosis are summarized in Table 1. The mean age was 50 +/- 16.1 years (range 22–68 years), with 6 male and 10 female patients. All patients had received at least one prior antidepressant trial; the most commonly used prior agents were escitalopram (*n* = 4), mirtazapine (*n* = 3), vortioxetine (*n* = 3), and amitriptyline (*n* = 2). Aripiprazole was initiated as augmentation therapy in 11 patients and as replacement monotherapy in 5 patients.

**Table 1:** Patient characteristics and CGI-I response by diagnosis (*n* = 16).

Diagnosis	*n*	Male/ Female	Mean age, years	Responders, *n* (%)
PPPD	9	3/6	52 ± 14	7 (78%)
Psychogenic dizziness	4	2/2	48 ± 19	3 (75%)
Vestibular migraine	2	1/1	45 ± 11	1 (50%)
Ménière's disease	1	0/1	61	1 (100%)
**Total**	**16**	**6/10**	**50** **±16**	**12 (75%)**

CGI-I, Clinical Global Impression-Improvement scale. One patient who discontinued due to extrapyramidal adverse effects (akathisia) is excluded from the response denominator (12/15 = 80.0%). PPPD, persistent postural-perceptual dizziness. Bold indicates the total/summary row.

**Figure 1:**
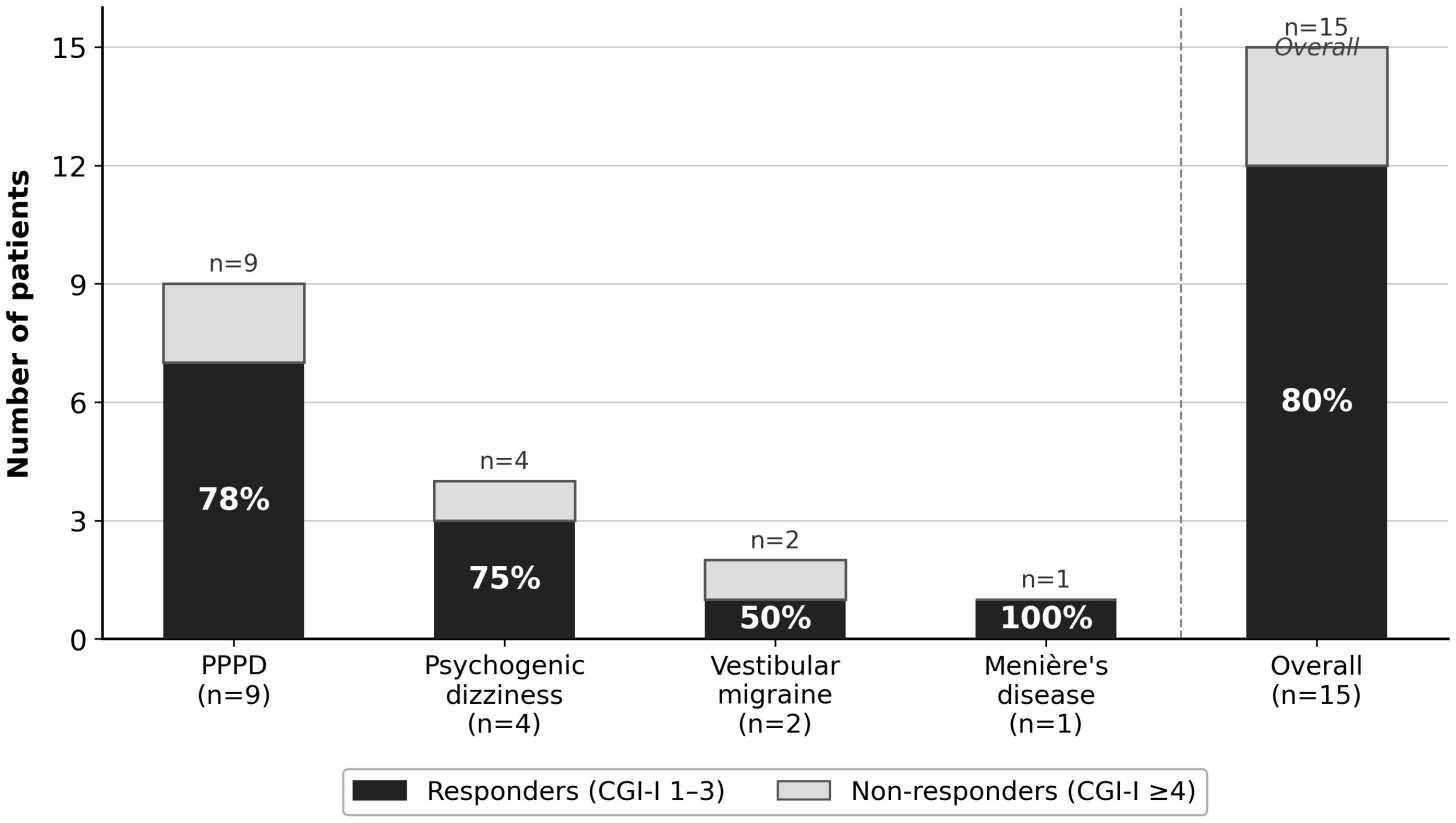
CGI-I response rates by diagnosis. Stacked bar chart showing the proportion of responders (CGI-I score 1–3, black bars) and non-responders (CGI-I score > = 4, white bars) for each diagnostic category and the overall cohort. One patient who discontinued due to extrapyramidal adverse effects is excluded (*n*=15 for Overall bar). Numbers above bars indicate total *n* per group. Percentages within black bars represent response rates. PPPD, persistent postural-perceptual dizziness; VM, vestibular migraine; MD, Meniere's disease. CGI-I, Clinical Global Impression-Improvement scale.

CGI-I response (score 1–3) was achieved in 12 of 16 patients (75.0%). Four patients (25.0%) were classified as non-responders (CGI-I >= 4). One patient discontinued aripiprazole due to extrapyramidal adverse effects (akathisia) and was excluded from the response denominator, yielding a response rate of 12/15 (80.0%) among patients who completed the treatment trial. Response rates by diagnostic category are shown in Figure 1: PPPD 7/9 (78%), psychogenic dizziness 3/4 (75%), vestibular migraine 1/2 (50%), and Meniere's disease 1/1 (100%). No other serious adverse events were recorded.

Pre- and post-treatment questionnaire data were available in five patients (Table 2). In all five evaluable cases, DHI scores decreased following aripiprazole treatment (range of improvement: 8-40 points). NPQ scores were available in four patients and showed improvement in all four. HADS anxiety and depression subscores also decreased in all evaluable patients, though the small sample size precluded formal statistical analysis. Foam posturography (closed-eye sway area on rectangular foam rubber, eyes closed) data with paired pre- and post-treatment measurements were available in all 16 patients. Pre-treatment closed-eye sway area on foam was 13.96 ± 8.69 cm^2^ (median 11.98, range 5.12–36.46); post-treatment closed-eye sway area was 13.22 ± 9.56 cm^2^ (median 10.51, range 4.91–42.18). Paired comparison (Wilcoxon signed-rank test) revealed no statistically significant change (median paired difference −0.02 cm^2^, mean paired difference −0.73 ± 6.38 cm^2^, p = 0.756). Although individual values showed substantial scatter, with 9 of 16 patients exhibiting absolute changes exceeding 20% of baseline in either direction, there was no systematic group-level shift: similar numbers of patients showed apparent improvement (*n* = 8, decreased sway area) and apparent worsening (*n* = 8, increased sway area). Foam closed-eye sway velocity showed a comparable pattern (pre 4.73 ± 1.94 cm/s, post 4.34 ± 2.04 cm/s, Wilcoxon p = 0.231). Notably, this absence of objective change occurred against a background of clear subjective improvement on CGI-I, DHI, and NPQ, consistent with prior reports of a dissociation between subjective and objective balance measures in PPPD pharmacotherapy (4).

**Table 2:** Pre- and post-treatment questionnaire scores in evaluable patients (*n* = 5).

Variable	Case 1	Case 2	Case 3	Case 4	Case 5
Diagnosis	Functional	PPPD	Functional	PPPD	PPPD
Age/Sex	39 M	22 M	57 F	45 M	41 M
Prior agent	ESC	MIR	ESC	AMI	VOR
Aripi. strategy	Add-on	Add-on	Add-on	Add-on	Add-on
DHI pre/post	44/6	88/72	52/30	30/20	58/18
NPQ pre/post	42/2	N/A	43/22	23/18	43/20
HADS-A pre/post	10/3	21/15	7/5	5/3	17/8
HADS-D pre/post	7/5	15/8	8/5	7/5	11/8
CGI-I	1	3	2	2	1

ESC, escitalopram; MIR, mirtazapine; AMI, amitriptyline; VOR, vortioxetine. Aripi., aripiprazole. DHI, Dizziness Handicap Inventory (higher scores = worse handicap). NPQ, Niigata PPPD Questionnaire (higher scores = greater symptom burden). HADS-A/D, Hospital Anxiety and Depression Scale, Anxiety/Depression subscale (score ≥8 indicates probable anxiety or depression). N/A, not assessed. CGI-I, Clinical Global Impression-Improvement (1=very much improved; 2=much improved; 3= minimally improved). Formal statistical testing was not performed given the small evaluable subset.

## Discussion

In this preliminary retrospective series of 16 patients with refractory dizziness, aripiprazole demonstrated a CGI-I response rate of 75.0% (80.0% among those who completed treatment), comparing favorably to the established 60-65% response rate of first-line SSRIs in PPPD (3, 4). This is particularly noteworthy given that all patients in this series had already failed at least one first-line antidepressant, representing a genuinely treatment-refractory population.

The pharmacological rationale for aripiprazole in refractory PPPD centers on its multimodal mechanism. Serotonin primarily modulates anxiety, norepinephrine regulates motivational drive, and dopamine underpins emotional stability and reward-related hedonic tone (5). Patients with refractory PPPD may exhibit a pattern of emotional dysregulation—characterized by affective instability and impaired motivational processing—that is not adequately addressed by serotonergic agents targeting anxiety and mood alone. Aripiprazole's D2 partial agonism normalizes dopaminergic tone bidirectionally (the ‘dopamine stabilizer' effect), while simultaneous 5-HT1A partial agonism reduces serotonergic overactivity and 5-HT2A antagonism provides anxiolytic-like modulation. This integrated trimodal action may explain its efficacy in cases where selective serotonergic strategies have been insufficient.

The response pattern across diagnostic categories deserves comment. PPPD showed a 78% response rate, consistent with the hypothesis that dopaminergic dysregulation is particularly relevant in this functional disorder. The high rate in psychogenic dizziness (75%) further supports this interpretation, as psychogenic dizziness shares with PPPD a strong component of emotionally-driven symptom amplification. The lower rate in vestibular migraine (50%) may reflect the heterogeneous pathophysiology of this condition, in which trigemino-vascular mechanisms may predominate over dopaminergic dysregulation in some patients.

The absence of posturographic change despite clear subjective improvement mirrors findings from the long-term PPPD pharmacotherapy study by Yagi et al. (4), published in Frontiers in Neurology Neuro-Otology section, which reported sustained improvements in DHI, HADS, and NPQ over 3 years with serotonergic antidepressants, while somatosensory hypersensitivity (measured by head-tilt perception gain) remained unchanged. Taken together, these findings suggest that pharmacotherapy—whether serotonergic or SDM—targets higher-order affective and perceptual-cognitive circuits, improving the subjective experience of dizziness, while low-level brainstem and spinal postural control mechanisms remain unaffected. Normalization of these objective balance parameters may require additional interventions such as vestibular rehabilitation or cognitive-behavioral therapy.

Regarding safety, one patient developed akathisia requiring discontinuation. This is consistent with the known pharmacology of aripiprazole at dopamine D2 receptors, though the low dose range used (1.5–3.0 mg/day) is associated with a substantially more favorable extrapyramidal side-effect profile than doses used in psychiatric indications (typically 10-30 mg/day). The remaining 15 patients tolerated aripiprazole without serious adverse events, supporting its acceptability at low doses in this clinical context.

Several important limitations must be acknowledged. First, the sample size (*n* = 16) is small, which substantially limits the statistical power and reliability of the response-rate estimates and precludes meaningful between-subgroup comparisons; the apparent differences in response rates across diagnostic categories should therefore be interpreted as descriptive only. Accordingly, the present findings should be regarded as hypothesis-generating rather than confirmatory, and this report is best characterized as a pilot investigation intended to inform the design and feasibility of future prospective controlled trials. Second, the retrospective uncontrolled design precludes causal inference; the observed improvement may reflect natural disease course, regression to the mean, or placebo effects rather than specific pharmacological action. Third, CGI-I is a clinician-rated assessment subject to expectation bias, particularly when the treating physician is also the rater. Fourth, questionnaire data were available in only five patients, preventing robust statistical analysis of patient-reported outcomes. Fifth, the diagnostic heterogeneity of the cohort limits subgroup conclusions. Sixth, data on prior receipt and adherence of vestibular rehabilitation, and the use of cognitive-behavioral therapy, were not systematically captured in this retrospective dataset, and formal cognitive-behavioral therapy was not available within our clinic setting; given that vestibular rehabilitation and cognitive-behavioral therapy are guideline-recommended non-pharmacological interventions for PPPD, the lack of structured documentation of these treatments before aripiprazole initiation is an important limitation, and the present efficacy estimates should be interpreted in light of this incomplete characterization of pre-aripiprazole non-pharmacological care. Finally, the single-center design may limit generalizability. Despite these constraints, this preliminary series provides clinically relevant data supporting further investigation and, combined with prior published case reports (6, 7), constitutes a sufficient evidence base to justify a prospective randomized trial of aripiprazole for refractory functional dizziness.

In conclusion, aripiprazole demonstrated preliminary efficacy and acceptable tolerability as second-line pharmacotherapy in a retrospective series of patients with refractory dizziness including PPPD. Its multimodal serotonin-dopamine mechanism may address affective dysregulation that is not adequately managed by serotonergic agents alone. Prospective controlled studies with standardized outcome measures are warranted to confirm these findings and establish the optimal dosing and patient selection criteria.

## Data Availability

The raw data supporting the conclusions of this article will be made available by the authors, without undue reservation.

## References

[1] Staab JP Eckhardt-Henn A Horii A Jacob R Strupp M Brandt T . Diagnostic criteria for persistent postural-perceptual dizziness (PPPD): consensus document of the committee for the classification of vestibular disorders of the Barany Society. J Vestib Res. (2017) 27:191–208. doi: 10.3233/VES-170622 29036855 PMC9249299

[2] Popkirov S Staab JP Stone J. Persistent postural-perceptual dizziness (PPPD): a common, characteristic and treatable cause of chronic dizziness. Pract Neurol. (2018) 18:5–13. doi: 10.1136/practneurol-2017-001809 29208729

[3] Min S Kim JS Park HY. Predictors of treatment response to pharmacotherapy in patients with persistent postural-perceptual dizziness. J Neurol. (2021) 268:2523–32. doi: 10.1007/s00415-021-10427-7 33544219

[4] Yagi C Kimura A Kai R Yamagishi T Ohshima S Izumi S . Long-term outcomes of pharmacotherapy in patients with persistent postural-perceptual dizziness. Front Neurol. (2025) 16:1566898. doi: 10.3389/fneur.2025.1566898 40177407 PMC11961416

[5] Stahl SM. Dopamine system stabilizers, aripiprazole, and the next generation of antipsychotics, part 2: illustrating their mechanism of action. J Clin Psychiatry. (2001) 62:923–4. doi: 10.4088/JCP.v62n1201 11780870

[6] Goto F Kaneda S Wasano K Okami K. A case of refractory dizziness effectively treated with the addition of aripiprazole to antidepressant therapy. Otolaryngol Case Rep. (2025) 34:100657. doi: 10.1016/j.xocr.2025.100657

[7] Goto F Kaneda S Wasano K Okami K. Brexpiprazole augmentation in antidepressant therapy for persistent postural-perceptual dizziness (PPPD): a case report. Otolaryngol Case Rep. (2025) 35:100664. doi: 10.1016/j.xocr.2025.100664

[8] Yagi C Morita Y Kitazawa M Nonomura Y Yamagishi T Ohshima S . A validated questionnaire to assess the severity of persistent postural-perceptual dizziness (PPPD): the Niigata PPPD Questionnaire (NPQ). Otol Neurotol. (2019) 40:e747–52. doi: 10.1097/MAO.0000000000002325 31219964 PMC6641087

